# Morphological abnormalities and cell death in the Asian citrus psyllid (*Diaphorina citri*) midgut associated with *Candidatus* Liberibacter asiaticus

**DOI:** 10.1038/srep33418

**Published:** 2016-09-15

**Authors:** Murad Ghanim, Somayeh Fattah-Hosseini, Amit Levy, Michelle Cilia

**Affiliations:** 1 Department of Entomology, Volcani Center, P.O. Box 6, Bet Dagnan 50250, Israel; 2 Boyce Thompson Institute for Plant Research, Ithaca, NY 14853, United States; 3 Plant Pathology and Plant-Microbe Biology Section, School of Integrative Plant Science, Cornell University, Ithaca, NY 14853, United States; 4 USDA Agricultural Research Service, Emerging Pests and Pathogens Research Unit, Ithaca, NY 14853, United States

## Abstract

*Candidatus* Liberibacter asiaticus (CLas) is a phloem-limited, gram-negative, fastidious bacterium that is associated with the development of citrus greening disease, also known as Huanglongbing (HLB). CLas is transmitted by the Asian citrus psyllid (ACP) *Diaphorina citri*, in a circulative manner. Two major barriers to transmission within the insect are the midgut and the salivary glands. We performed a thorough microscopic analysis within the insect midgut following exposure to CLas-infected citrus trees. We observed changes in nuclear architecture, including pyknosis and karyorrhexis as well as changes to the actin cytoskeleton in CLas-exposed midgut cells. Further analyses showed that the changes are likely due to the activation of programmed cell death as assessed by Annexin V staining and DNA fragmentation assays. These results suggest that exposure to CLas-infected trees induces apoptotic responses in the psyllid midgut that should be further investigated. Understanding the adaptive significance of the apoptotic response has the potential to create new approaches for controlling HLB.

*Candidatus* Liberibacter asiaticus (CLas) is a phloem-limited, Gram-negative, fastidious bacterium that is implicated in causing the most serious disease of citrus, citrus greening disease, also referred to as Huanglongbing. The bacterium is transmitted by the Asian citrus psyllid (ACP), *Diaphorina citri* Kuwayama^1^,^2^. The ACP and HLB have spread to most citrus growing regions around the world. In the United States, HLB has decimated Florida’s annual nine billion dollar industry, and the pathogen and vector are emerging into other citrus growing areas. *Candidatus* Liberibacter solanacerarun (CLso) is another phloem-limited, Gram-negative, unculturable bacterium transmitted by psyllids. This bacterium has been associated with serious diseases of tomatoes, potatoes and other solanaceous crops, especially with the important potato disease called zebra chip, which has caused economic losses by reducing yield and quality of potato crops. In potatoes, CLso is transmitted by the potato/tomato psyllid *Bactericera cockerelli*. The bacterium has also been associated with vegetative disorders in fennel, celery and carrots^3^ and is transmitted by various psyllid species, including *Trioza apicalis* in Europe and *Bactericera trigonica* (BT) in the Middle East. Current management options for HLB and the diseases caused by CLso are limited and rely heavily on the application of chemical insecticides for limiting psyllid populations^4^,^5^,^6^,^7^,^8^,^9^,^10^. The use of insecticides by the Florida citrus industry has drastically increased the cost of business and placed serious economic strain on the citrus economy. Immediate and sustainable measures for controlling the disease are urgently needed. As an alternative, disrupting bacterial transmission by psyllid vectors, rather than controlling the insects, represents an improved disease management strategy.

CLas and CLso are transmitted by psyllids in a persistent manner. In the case of CLas, the bacterium has been detected in various ACP organs, including the salivary glands, hemolymph, filter chamber, midgut, fat and muscle tissues, and ovaries^11^. Previous research has shown that to be efficiently transmitted by adults, CLas has to be acquired in the nymphal stage and reach sufficient amounts for transmission. Several parameters for CLas interaction with ACP, including acquisition, retention, latent period and transmission have been studied^12^. In lab and field conditions, acquisition by nymphs ranged from 60 to 100%, whereas acquisition by adults only reached 40% after weeks of feeding on CLas-infected plants^12^. One year after psyllid inoculations, successful transmission by individual ACP ranged from 4 to 10%, whereas groups of 100 or more ACP transmitted the pathogen with 88% efficiency^12^. Due to the low rate of pathogen acquisition and long time period required for successful inoculation by adult ACP, it was not possible to determine the latent period^12^. These results indicated that ACPs which acquire the CLas as adults are poor vectors of the pathogen compared with adults that acquired the pathogen as nymphs, suggesting that bacterial multiplication in the psyllid may be essential for efficient transmission^13^, although replication of the bacterium in insects has not been conclusively shown. The interactions and parameters for acquisition, transmission and retention of CLso by *B. cockerelli* have also been investigated, but with fewer details as compared to CLas. One study showed that increasing the latency period increased the chance the bacteria would reach the salivary glands and be transmitted, while a two week latent period was a prerequisite for transmission by adults^14^. A certain amount of the bacterium has to be present in the salivary glands to be transmitted^14^. Transmission rates of the CLas and CLso probably depend on both the titer of bacteria in insect tissues and on the barrier to transmission. The molecular interactions and biochemical pathways that determine barrier specificity and acquisition of CLas are not known.

Circulative transmission of CLas by the ACP starts with ingestion of the bacteria into the insect from the phloem of infected trees by the insect’s piercing, sucking mouthparts. The midgut is the first barrier where the bacterium must breach before acquisition into the hemolymph. Midgut acquisition of other vector-borne pathogens has been suggested to involve specific receptors that aid these pathogens in crossing the midgut barrier to the hemolymph^15^,^16^,^17^. For non-propagative viruses such as poleroviruses, luteoviruses and begomoviruses transmitted by aphids and whiteflies, respectively, virus acquisition involves vesicle trafficking and transport within the cytoplasm of the polarized gut epithelial cells. In this manuscript, phenotypes of nuclear fragmentation and cellular abnormalities were observed in midguts dissected from CLas-exposed insects. Further analyses showed that these cellular changes were associated with expression of markers for programmed cell death as well as DNA degradation. These results support the hypothesis that the ACP mounts a specific immune response in the midgut when exposed to CLas-infected phloem sap associated with CLas acquisition. It is possible that this immune response is related to the inability of adults to efficiently acquire and transmit CLas^12^,^13^.

## Results

### CLas is acquired into the ACP midgut

**Figure 1:**
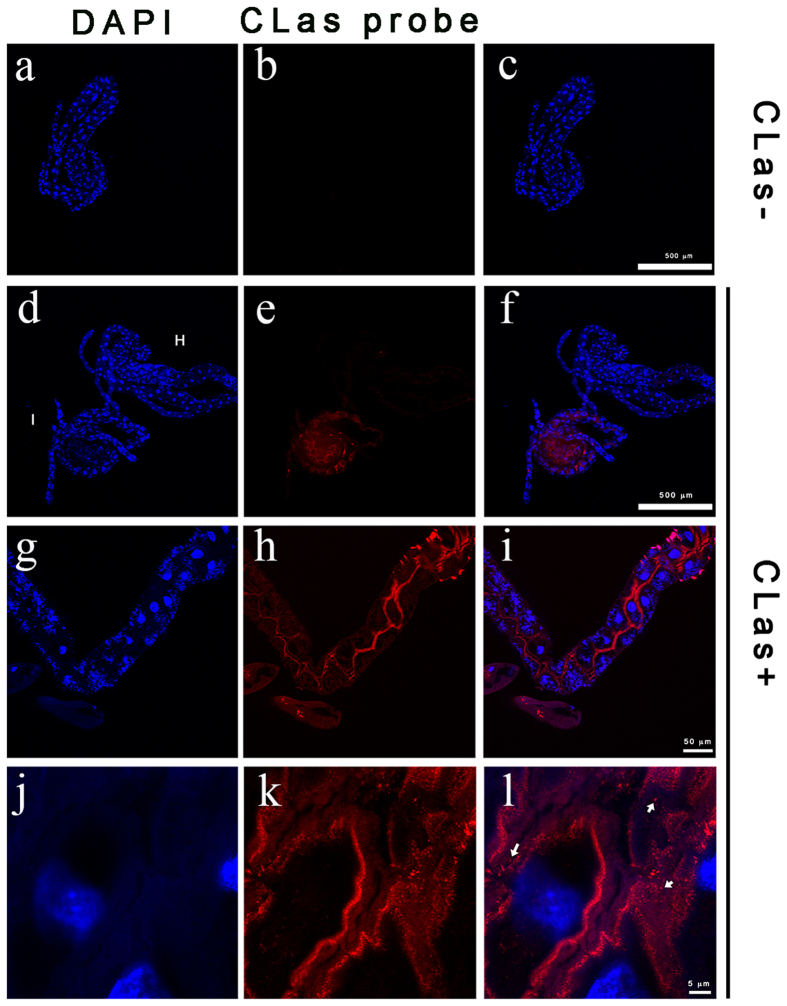
Fluorescence *in situ* hybridization (FISH) using a CLas probe (red) and DAPI counterstaining (blue) of epithelial cell nuclei in midguts dissected from *Diaphorina citri* adults. The left column shows DAPI staining only, the middle column show the CLas probe only, and the right column shows a merged image. Panels a–c show guts from insects reared on healthy trees (non-exposed) show no detectible CLas-FISH signal. Panels d–f show a non-exposed gut (NE) and a CLas-exposed gut (I) in the same image, showing the specificity of the CLas-FISH signal in CLas-exposed guts. The CLas probe reveals CLas within the midgut epithelial cells and the filter chamber. Midguts from CLas-exposed insects at higher magnification show FISH signal on the cell membranes (**h,i**) and in a punctate pattern with 0.5 μm diameter in the gut (**j–l**).

**Figure 2:**
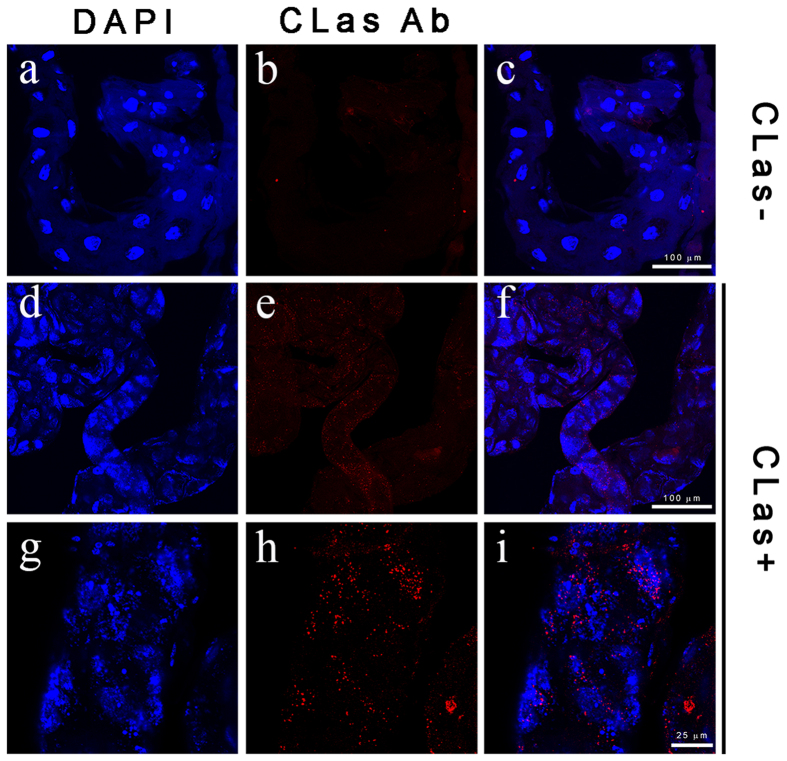
Immunostaining of CLas using anti-CLas OmpA antibody in dissected midguts from insects reared on healthy trees (non-exposed, (a–c)) and CLas-exposed *Diaphorina citri* adults (d–i). Left panels show DAPI signal, middle panels show OmpA immunostaining signal, and right panel contained merged images. Immunostaining shows primarily intracellular CLas localization.

The acquisition of CLas by ACP reared on CLas-infected plants was studied using several methods. First, using a Cy3-labeled DNA probe targeting CLas 16S ribosomal RNA sequence, we used FISH to visualize CLas in the guts. CLas was clearly visualized in the midgut and the filter chambers of psyllids reared on CLas-infected trees. Out of a total of 89 CLas-exposed psyllids examined, CLas was detected in 40% of the insects. No signal was detected in the guts of psyllids that were reared on healthy trees under the same conditions (Fig. 1). Upon examination of the midguts under higher magnification, the CLas FISH probe was detected as small dots with diameter of about 0.5 μm in the gut lumen. The signal also accumulated along the midgut cell membranes (Fig. 1). CLas acquisition was further verified using immunolocalization with an anti-CLas OmpA antibody. The CLas OmpA signal appeared as a punctate pattern of dots with a diameter smaller than 1 μm, which localized inside the gut cells. CLas OmpA was only detected in psyllids reared on CLas-infected trees and not in psyllids reared on healthy trees (Fig. 2). Immunolocalization of CLas was widespread in the gut cells, and, unlike the FISH detection, did not accumulate along the brush border membranes (Fig. 2). Finally, the acquisition of CLas in psyllids that were reared on infected plants was also confirmed using quantitative real-time PCR (data not shown).

### Midguts from CLas-exposed insects show abnormalities in gross morphology and nuclear architecture, including pyknosis and karyorrhexis

**Figure 3:**
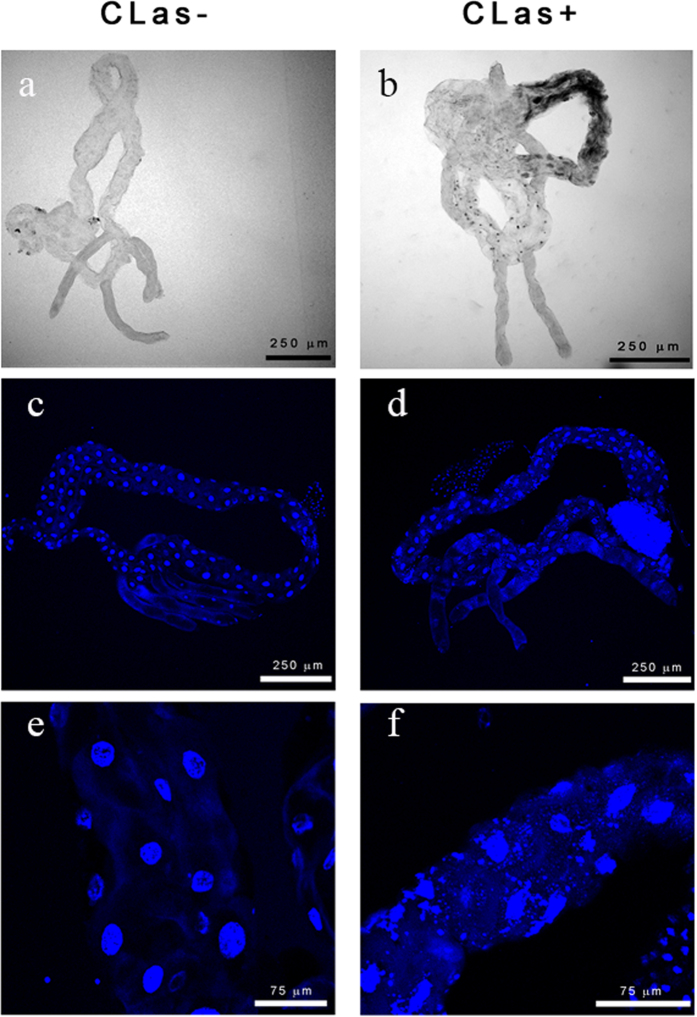
Microscopic analysis of cellular and morphological abnormalities are observed in midguts dissected CLas-exposed *Diaphorina citri* adults. Light micrograph of a gut dissected from an insect reared on a healthy, CLas-free citrus tree (non-exposed, (**a**)) shows normal gut morphology. Light micrograph of a representative gut dissected from a CLas-exposed insect shows dark spots, an irregular diameter around the midgut and extensive melanization (**b**). Low magnification images of representative insects reared on healthy trees or (**c**) and CLas-infected trees (**d**). Midguts show nuclear abnormalities in the CLas-exposed insects. High magnification images of midguts dissected from insects reared on healthy trees (non-exposed, (**e**)) show uniform and round epithelial cell nuclei. In contrast, CLas-exposed midgut epithelial cell nuclei are irregular in shape, size and fragmented throughout the cytoplasm (**f**).

**Figure 4:**
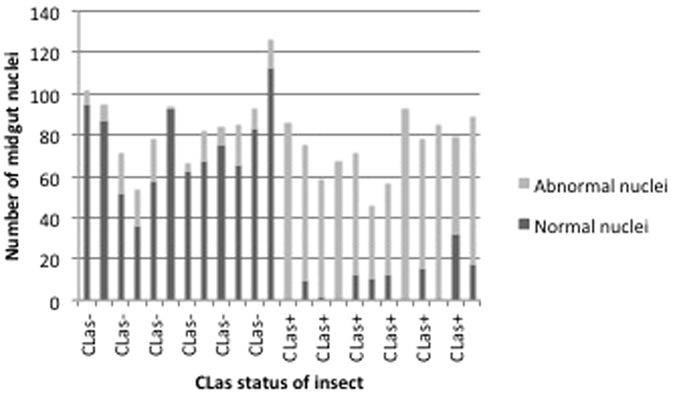
Quantification of normal and abnormal nuclei in midgut epithelial cells of psyllids exposed to healthy and CLas-infected trees. Insects reared on healthy trees show a very low number of abnormal nuclei in contrast to insects reared on CLas-infected trees, which show a high number of abnormal nuclei.

In the midguts from CLas-exposed ACP, we observed abnormal continuity of the midgut diameter as compared to guts from insects reared on healthy trees. We observed the formation of dark necrotic portions and necrotic spots that were not observed in guts from pysllids reared on healthy trees (Fig. 3). To study the nature of the necrotic phenotypes in more detail, we stained guts dissected from CLas-exposed or non-exposed adult insects with DAPI to observe nuclear structures and overall gut cell morphology. In midguts from CLas-exposed psyllids, DAPI stained nuclei showed clear evidence of pyknosis, that is the shrinkage of the nucleus due to irreversible chromatin condensation (Figs 1, 2 and 3). The majority of the pyknotic nuclei had lost their normal elliptical structure (Figs 1, 2 and 3). Other nuclei showed evidence of karyorrhexis, which is the fragmentation of the nucleus with the irregular distribution of chromatin throughout the cell. Many irregularly shaped and spaced punctate regions of chromatin were observed outside of the nucleus (Fig. 3). Nuclei from psyllids reared on healthy trees appeared regularly dispersed in the cells and of uniform shape, size and DAPI staining efficiency. Quantification of this effect showed that nuclei fragmentation in the midguts was observed in ~65% of the CLas-exposed adult psyllids reared on CLas-infected plants, but only in 12% of adult ACPs reared on healthy trees (Fig. 4). Notably, some of the abnormal phenotypes observed after DAPI staining occurred in guts of CLas-exposed insects that did not exhibit a positive FISH signal for CLas.

### CLas-exposed psyllid midgut cells undergo apoptosis

**Figure 5:**
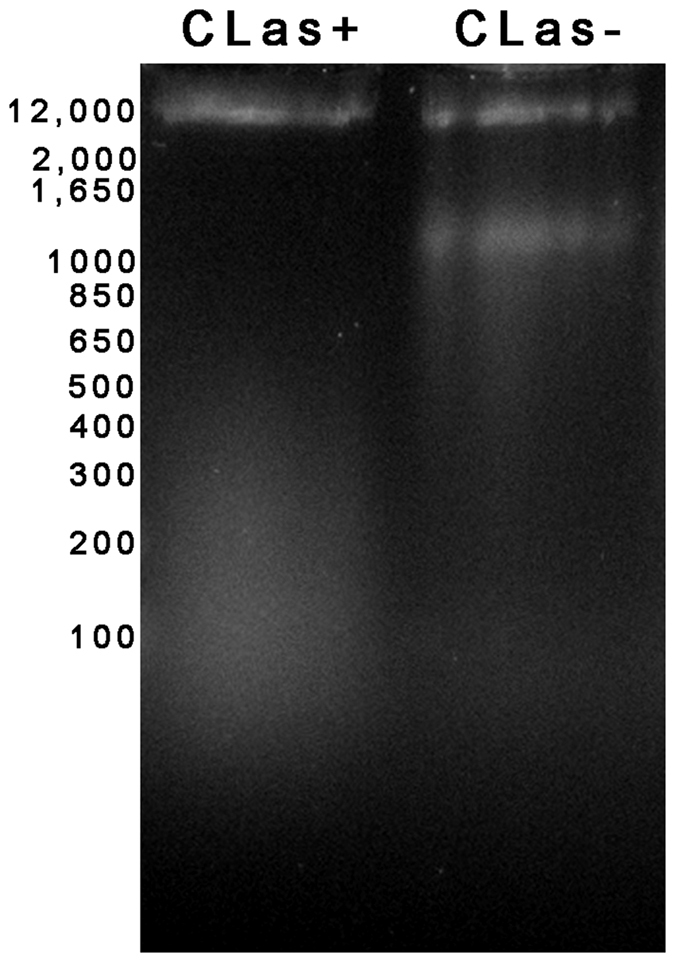
Assessment of DNA integrity following extraction from midguts using agarose gel electrophoresis. The DNA extracted from CLas-exposed insects (CLas+) shows an enrichment of lower molecular weight DNA fragments as compared to insects reared on healthy trees (non-exposed).

**Figure 6:**
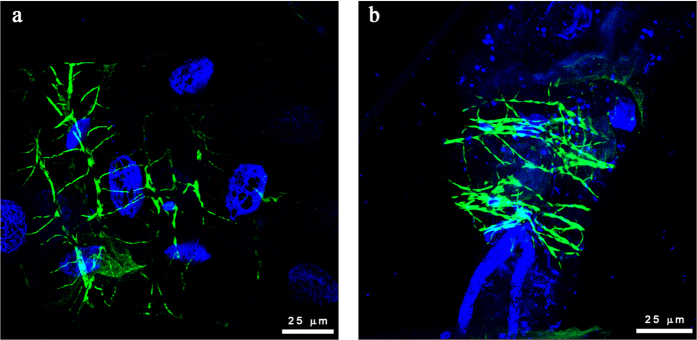
A comparison of the structure of the actin cytoskeleton in adult *Diaphorina citri* midguts dissected from insects reared on healthy or CLas-infected citrus trees using phalloidin staining. CLas-exposed insects show disrupted actin filaments (**b**) as compared to a more organized structure of filaments in the midguts from insects reared on healthy trees (**a**). A qualitative analysis showed filaments in the CLas-exposed midgut samples to connect to one another at irregular angles, appear shorter in places, and thicker in diameter.

Nuclear pyknosis and karyorrhexis are hallmark signatures of cells undergoing cell death, either via apoptosis or necrosis. Many of the signaling events that occur in apoptosis and necrosis are shared, including a breakdown of the integrity of the genome DNA and organization of the actin cytoskeleton^18^. To investigate the integrity of the genome DNA in apoptotic cells, we isolated DNA from CLas-exposed and non-exposed insect guts and measured the DNA integrity using agarose gel electrophoresis. DNA extracted from midguts both CLas-exposed and non-exposed psyllid colonies displayed a clear high molecular weight band greater than 12 Kb. The non-exposed insects that were reared on healthy trees showed an additional band with a size of approximately 1500 bps, but this band was not present in samples from CLas-exposed insects (Fig. 5). Instead, DNA extracted from the guts of CLas-exposed insects contained a high level of mixed-size, smaller DNA fragments, less than 500 bp. Similarly, the actin cytoskeleton appeared unorganized and highly disrupted in CLas-exposed midguts as compared to those dissected from insects reared on healthy plants (Fig. 6). These results strongly support the hypothesis that cell death is occurring in CLas-exposed midguts; however, they do not distinguish between apoptosis and necrosis.

**Figure 7:**
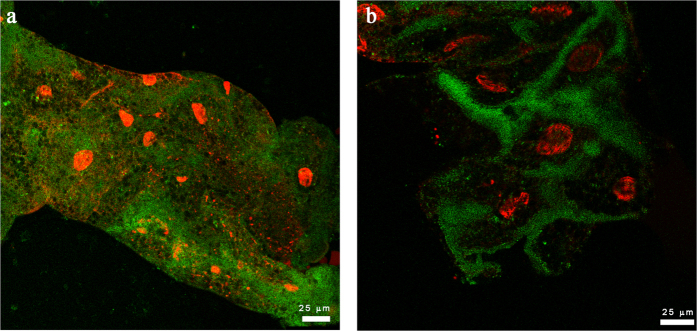
Apoptosis assay using Annexin V visualization in adult *Diaphorina citri* midguts reared on healthy or CLas-infected citrus trees. Nuclei are counterstained using propidium iodide (red). Midgut samples from insects reared on healthy trees showed either no Annexin V staining or a diffuse signal distributed throughout the cytoplasm (**a**). In contrast, CLas-exposed samples (**b**) showed Annexin V binding to the cell membranes, indicating the epithelial cells are undergoing apoptosis in response to exposure to CLas-infected trees.

To determine whether CLas-exposed midguts were undergoing necrosis or apoptosis, we stained midgut cells of psyllids that were reared on either healthy or CLas-infected trees using Annexin V. Phosphatidylserine (PS) moieties, which are recognized by Annexin V, are usually present only on the inner side of the cell membrane. In cells undergoing apoptosis, but not necrosis, PS is translocated to the outer cell membrane resulting in the ability to stain PS moieties and to detect apoptotic cells using fluorescently labeled Annexin V. The specificity of the PS translocation and PS-Annexin V interaction results in staining of the cell membrane in cells undergoing apoptosis^19^. In non-exposed adult psyllid guts reared on healthy plants, Annexin V showed two distinct staining patterns, either a diffused, non-specific signal distributed throughout the cytoplasm (Fig. 7) or no signal at all (not shown). In CLas-exposed guts, the signal localized to the gut cell membranes (Fig. 7), indicating these cells were undergoing apoptosis and not necrosis.

### Apoptosis induction by CLas is specific to the midgut cells

**Figure 8:**
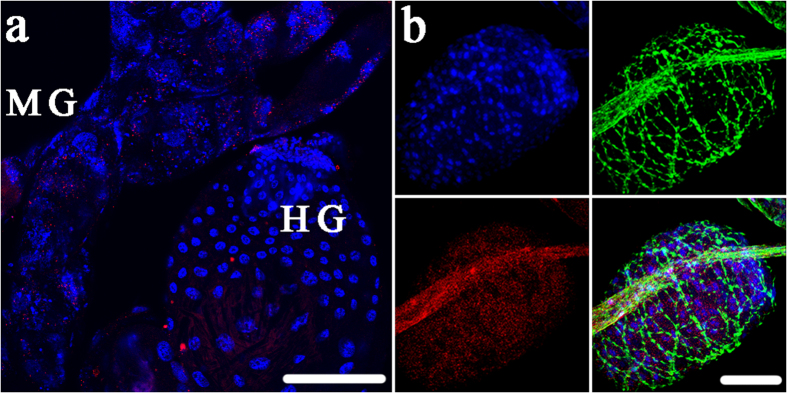
Differential effect of CLas on nuclear morphology and cytoskeleton organization in adult *Diaphorina citri* guts and oocytes. (**a**) In CLas-exposed insects, changes in nuclear morphology were clearly visualized in midgut cells (MG), but not hindgut (HG). Scale bar is 100 μm. (**b**) Differential effect of CLas on nuclear morphology and cytoskeleton organization in adult *Diaphorina citri* oocytes. CLas is also visualized by immunolocalization (red) using an anti-OmpA antibody. No changes in nuclear morphology as visualized by DAPI staining (blue) or actin filaments (green) in CLas-exposed insects were observed in oocytes dissected from CLas-exposed insects. Scale bar is 50 μm.

To determine whether the apoptosis we observed is specific to the midguts or a more generalized response in the CLas-exposed psyllids, we analyzed CLas localization and the development of apoptosis in additional portions of the digestive system of ACP that were reared on CLas-infected trees (Fig. 8a). In the guts, both the presence of CLas and the development of apoptosis were restricted to the midgut. In the hindgut of CLas-exposed insects, we did not observe CLas localization in any of the analyzed specimens. Cell and nuclear disruptions were also not observed, as determined by DAPI staining (Fig. 8a). A similar analysis was performed on oocytes dissected from females reared on CLas-infected plants (Fig. 8b). In the oocytes, we detected the presence of CLas using FISH, but DAPI staining did not show any nuclear fragmentation, suggesting that the cells in the eggs did not undergo apoptosis in response to CLas (Fig. 8b). These data show that CLas is present in tissues other than the midgut; however, CLas-induced apoptosis was only observed in the midgut (Figs 1, 3, 4, 7 and 8).

## Discussion

Evidence shows the relationship between insect vectors and plant pathogens is along a continuum, where at one end you have a competitive, host-pathogen relationship and at the other end, you have a mutualistic relationship. Some, but not all, plant pathogens induce apoptosis in the gut of their insect vectors, suggesting that apoptosis is specific response in a given vector-pathogen system, and will not occur in all such interactions. At the ultimate level, apoptosis in vector tissues, among other cellular and immune responses, may be selected for in vector-plant pathogen pairs where the plant pathogen is also a pathogen of the insect vector. Along this line of reasoning, it is intriguing that CLas (or the CLas-infected plant) induces apoptosis in the midgut of its psyllid vector and CLso does not. At the molecular level, although the genomes of CLso and CLas are similar, they are not identical. There might be a role for CLas/CLso- microbiota interactions in the guts of the insect vector in inducing apoptosis, and these interactions should be further investigated. The host plants of CLas and CLso are different, and the insects are responding not just to the pathogen, but to the infected plant phloem sap. Even among different citrus varieties there is a wide variation of HLB symptoms expressed and the impact of *in planta* symptom expression on vector tissues and behavior is not known. These factors may all play a role in why two closely related plant pathogens, CLas and CLso, appear to have different effects on their insect vectors.

An understanding of whether the midgut cells are responding to CLas using apoptosis or necrosis is key to our understanding of the relationship between CLas and its insect vector, where the former response would suggest the ACP cells are active participants in their own demise using the same cellular pathways reserved for normal immune system regulation and development and the latter would suggest a direct response to cell membrane damaging agents leading to cell swelling and lysis. Quantification of nuclear disruption showed higher levels in CLas-exposed midguts (65%) but some also occurred in the midguts of non-exposed insects reared on healthy citrus trees, suggesting that it is a process that may occur at a low frequency in the adult midgut as part of the maintenance of normal gut homeostasis. These interpretations are consistent with apoptosis, rather than necrosis, and is further supported by the Annexin V staining and DNA integrity assays.

Our data show that the level of apoptosis in the midgut is enhanced in CLas-exposed insects. The apoptotic responses observed in the midguts were specific to this tissue, suggesting induction of apoptosis is related to acquisition of CLas by the vector. It is pertinent to note previous work has shown that ACP that acquire CLas as adults are inefficient vectors^12^, which raises the question as to whether the apoptotic response we observed is correlated or causal to the inability of adults to efficiently become competent for transmission if the bacteria are acquired as adults. An alternative hypothesis could be that apoptosis in the midgut serves a protective role in regulating the passage of pathogenic microorganisms such as CLas into the insect hemolymph. Unlike CLas, CLso does not seem to induce apoptosis in the guts of potato psyllids^20^. Since CLas transmission by adult ACP (independent of the developmental stage at which the insect acquires the bacteria) is less efficient than the transmission of CLso by the potato psyllid, we hypothesize that the CLas-induced apoptosis in the midgut is an adaptive, targeted ACP immune response for destroying cells containing the bacteria. This response serves to limit the acquisition and transmission efficiency of CLas by the ACP. This hypothesis is also in line with the published data showing a minimal effect of CLas on the life history traits of the ACP, including longevity and fecundity^21^. CLas has a profound effect on the expression of proteins involved in propionate metabolism and the production of short-chain fatty acids in the ACP^22^. In human cells, short-chain fatty acids have been shown to induce autophagy as an adaptive strategy to slow the progression of mitochondria-mediated cell death^23^, and it is possible that psyllids are using a similar strategy to cope with CLas.

The observation of apoptosis in CLas-exposed insects where CLas was not detected and at a lower level in the midguts of insects reared on healthy citrus trees suggests that the phenotype could be mediated by another commensal microorganism residing in the midgut tissues and enhanced by the presence of CLas. Alternatively, the nuclear abnormalities we observed could be induced by the infected plant and not directly by the ingested or acquired CLas. The nuclear pyknosis and karyorrhexis phenotypes were weaker in CLas-exposed psyllids that did not harbor detectable levels of CLas as measured by FISH or OmpA immunolocalization as compared to insects which showed a CLas-FISH or OmpA antibody signal, but these data were not quantified here. It is quite possible that insects showing intermediate phenotypes for pyknosis and karyorrhexis were indeed CLas-positive, although the bacterial titer was below the limit of FISH or OmpA antibody detection.

ACP genome and transcriptome sequencing^24^ and annotation reveals that the vector is tooled with the genes to execute the two well-defined apoptosis pathways^25^. The extrinsic pathway is initiated by transmembrane receptors belonging to the TNFR superfamily. It is activated by “death ligands” such as TNF-α, TRAIL, and FasL^26^. In contrast, the intrinsic apoptosis signaling pathways involve non-receptor mediated events that act intracellarly to induce changes in the inner mitochondrial membrane that results in the loss of mitochondrial permeability and mitochondrial membrane potential. The primary regulators of the intrinsic pathway are proteins belonging to the Bcl-2 family^25^. Caspases, which belong to the family of cysteinyl proteases, are the major components of the apoptotic machinery^27^. The ACP genome encodes the major apoptosis-related gene families including *apoptosis inducing factors*, Bcl2s, caspases, DNA fragmentation factor alpha (DFFA) and inhibitors of apoptosis (IAPs).

The role of apoptosis in insects that transmit plant and animal pathogens is not well studied, but new research shows apoptosis can play highly diverse roles in the vector-pathogen relationship. Huang and colleagues recently demonstrated that *Rice ragged stunt virus* (RRSV) induced apoptosis in the salivary glands of its vector, the brown planthopper *Nilaparvata lugens* Stål. Interfering with apoptosis inhibited RRSV transmission, suggesting that the establishment of apoptosis is critical in maintaining the relationship between the virus and its insect vector^28^. In contrast, the animal-infecting arbovirus West Nile virus has been shown to induce apoptosis in the midgut and salivary glands of its mosquito vector *Culex pipiens pipiens*^29^. O’Neill and colleagues tested the role of apoptosis as an antiviral defense to Sindbis virus (SINV) infection in *Aedes aegypti* mosquitoes^30^. Mosquitoes were infected with a strain of SINV expressing the proapoptotic gene reaper from *Drosophila*. Insects infected with the mutant virus showed delayed midgut infection and virus replication as well as reduced virus titer in the saliva. In addition, they observed a strong negative selection against reaper in virus isolated from individual insects^30^. Taken together, these studies show that apoptosis is a potent antiviral defense response that is actively targeted by vector-borne pathogens for successful acquisition and transmission. Its role in vector-pathogen relationships for bacterial pathogens remains an open question. However our work here adds to the accumulating body of work that shows viruses and bacteria^31^ have developed an array of mechanisms to either escape dell death or to manipulate it for their own benefit during their transmission to the next host and reveals the apoptotic midgut as a potential interdiction point in the acquisition of CLas by the ACP.

Practically speaking, how may an understanding of apoptosis and the psyllid immune response to CLas be leveraged to control the spread of HLB? One approach would be to develop interdiction molecules that interfere with apoptosis in the midgut directly and a second approach involves gene silencing of apoptosis related genes in the psyllid. Gut binding peptides that impede the uptake of plant viruses by aphid vectors have been developed^16^ and the discovery of such peptides that interfere with CLas acquisition has been a major and successful focus of the nuPsyllid project, a multidisciplinary project jointly funded by the US Department of Agriculture and the Florida Citrus Research and Development Foundation (CRDF, http://citrusrdf.org/nupsyllid-page-2) and Citrus Greening Solutions (www.citrusgreening.org), a second project funded by the US Department of Agriculture Specialty Crops program on citrus greening. The second approach, RNAi, is now widely discussed for plant pest management^32^,^33^. The psyllid genome encodes the machinery for RNAi and silencing psyllid genes using RNAi has been demonstrated^34^,^35^,^36^,^37^,^38^. Field deployment of RNAi strategies could be accomplished via virus induced gene silencing in plants using viral vectors^35^ or perhaps via newly discovered psyllid-infecting viruses^39^,^40^. According to the CRDF^41^, an estimated time frame for development of such strategies would be one year of lab research, and two to five years of field-testing on the impact of reducing ACP populations and/or HLB spread. Eventual field deployment, at least in the United States, will require USDA and EPA approval, and these approvals may take an additional six months to a year^41^. Continued research, development and public education on the safety and efficacy of the tools in the precision HLB management tool box will be vital to save the citrus industry worldwide.

## Materials and Methods

### Insect, plant and CLas materials

ACP populations used in this study were originally obtained from Dr. David Hall at the USDA ARS Horticultural Research Lab, Fort Pierce, Florida. In the Cilia lab at the USDA ARS Robert W. Holley Center in Ithaca, NY, synchronized populations were maintained on Madam Vinous sweet orange (Citrus sinensis (L. Osbeck) grown from certified clean seed obtained from the National Clonal Germplasm Repository for Citrus and Dates in insect-proof cages inside environmentally controlled growing chambers with a twelve hour photoperiod. CLas-infected plants were obtained using ACP inoculation and CLas-exposed ACP were maintained under the same growth conditions as the insects reared on healthy plants. In this paper, we will refer to these insects as non-exposed insects. CLas status of the insects was verified by qPCR^42^.

### Psyllid gut dissection and staining with DAPI, PI, and phalloidin to visualize nuclear architecture and actin

Staining of psyllid cells using a variety of cell biology probes was performed to assess the impact of CLas-exposure on adult ACP midgut and other tissues. Guts and reproductive organs were dissected from adults in 1× phosphate buffered saline (PBS) under a dissecting stereomicroscope by using depressed glass wells and fine entomological needles. After a sufficient number of replicate tissue samples were dissected (20 or more), the tissues were washed 2–3 times with 1× PBS and subjected to the staining methods described below.

The stain 4′,6-diamidino-2-phenylindole (DAPI) was used to visualize the nuclei in midgut cells. CLas-exposed and non-exposed guts were dissected and immersed in a DAPI solution at 0.1 mg/ml in 1× PBS (pH 7.2) and were mounted on microscope slides, covered with coverslips and visualized using a Leica TCS SP5 laser scanning confocal microscope. Propodium iodide (PI) staining was also used to visualize nuclei. For PI staining, the DAPI procedure was followed except that a working solution of 1 μg/ml in 1× PBS was used^19^. Phalloidin was used to stain actin filaments in CLas-exposed and non-exposed guts. Dissected gut tissues were fixed in 4% paraformaldehyde in 1XPBS for 1 h. The guts were then incubated with 0.5 mg/ml FITC-conjugated phalloidin (Sigma) diluted 1:1000 in PBST for 30 min, washed with PBS containing 0.05% Tween 20 (PBST), mounted in PBST on microscope slides, covered with cover slips, and viewed under a Leica TCS SP5 laser scanning confocal microscope.

### Fluorescent *in situ* hybridization (FISH) for CLas detection

FISH was used to visualize the distribution of CLas in CLas-exposed ACP. Midguts from CLas-exposed and non-exposed ACPs were dissected in 1× PBS (pH 7.2) inside depressed glass well, fixed in Carnoy’s fixative (chloroform-ethanol-glacial acetic acid [6:3:1, vol/vol] formamide) containing 10 pmol fluorescent probe/ml. The CLas probe sequence used in this study is 5′Cy3-GCCTCGCGACTTCGCAACCCAT-3′. Nuclei in the midgut were counterstained with DAPI. After hybridization overnight and DAPI staining, the gut samples were transferred to slides, mounted whole in hybridization buffer and viewed using confocal microscopy. For each analysis, at least twenty midguts were viewed. Optical confocal sections (100 μm thick) were acquired from a randomly selected subset of the gut specimens for better visualization of the signal.

### Immunolocalization of CLas

As an alternative to FISH, immunolocalization was used to visualize CLas in CLas-exposed insect tissues. CLas-exposed and non-exposed ACP midguts were dissected on glass microscope slides under a dissecting microscope in 1× PBS and fixed in 4% paraformaldehyde for 30 min at room temperature. Midguts were permeabilized by adding 0.1% Triton X-100 for 30 min at room temperature. The midguts were washed three times with PBST, blocked for 1 h at room temperature with blocking buffer (PBST with 1% [w/v] bovine serum albumin), and incubated overnight at 4 °C with anti-CLas outermembrane porin (OMP) polyclonal primary antibody raised in rabbit (Abnova, PAB15989). Midguts were then washed three times with PBST and incubated with goat anti-rabbit secondary antibody conjugated to Cy3 (Jackson Laboratories) for 1 h at room temperature. Midguts were washed again three times with PST, mounted in 1× PBS containing 0.1 mg/ml DAPI, covered with a coverslip, sealed with nail polish, and viewed using confocal microscopy.

### Annexin V cell death assay

To test whether cells were undergoing apoptosis or necrosis, annexin staining^19^ was performed using the Annexin V-FITC apoptosis detection kit (Abcam AB14085) following the manufacturer’s instructions with the following modifications: after the midguts were dissected in 1× PBS as described above, they were chopped into small pieces using entomological needles to allow better penetration of the reagents into the gut cells. After chopping, the guts were fixed with 4% paraformaldehyde in 1× PBS (pH 7.2) for 5 min. The guts were then washed with binding buffer and incubated with Annexin V-FITC and PI for 3–4 h. The guts were again washed with binding buffer three times, mounted on microscope slides and viewed using confocal microscopy.

### DNA degradation assay

To test for integrity of the genomic DNA in apoptotic cells, the DNA was extracted from 50 CLas-exposed and non-exposed ACP midguts using the blood/tissue DNA extraction kit (Qiagen). DNA was run on 2% agarose gels, stained using Gel Red (Biotium) and visualized using UV light.

## Additional Information

**How to cite this article**: Ghanim, M. *et al*. Morphological abnormalities and cell death in the Asian citrus psyllid (*Diaphorina citri*) midgut associated with *Candidatus* Liberibacter asiaticus. *Sci. Rep.* **6**, 33418; doi: 10.1038/srep33418 (2016).
